# Potentially modifiable factors associated with non-adherence to phosphate binder use in patients on hemodialysis

**DOI:** 10.1186/1471-2369-14-208

**Published:** 2013-10-03

**Authors:** Maria Tereza Silveira Martins, Luciana Ferreira Silva, Angiolina Kraychete, Dandara Reis, Lidiane Dias, Gabriel Schnitman, Lívia Oliveira, Gildete Barreto Lopes, Antonio Alberto Lopes

**Affiliations:** 1 Programa de Pós-Graduação em Medicina e Saúde, Universidade Federal da Bahia, Salvador, BA, Brazil; 2 Departamento de Ciências da Vida, Universidade do Estado da Bahia, Salvador, Bahia, Brazil; 3 Instituto de Nefrologia e Diálise, Salvador, Bahia, Brazil; 4 Núcleo de Epidemiologia Clínica e Medicina Baseada em Evidências, Salvador, Bahia, Brazil; 5 Departamento de Medicina e Apoio Diagnóstico da Faculdade de Medicina da Bahia, da Universidade Federal da Bahia, Salvador, Bahia, Brazil

**Keywords:** Hemodialysis, Phosphate binder, Adherence

## Abstract

**Background:**

Despite the evidence that phosphate binder (PB) is associated with improved outcomes many hemodialysis patients do not adhere to prescribed PB regimen. Therefore, barriers to PB adherence should be identified and eliminated. The purpose of this study was to evaluate PB adherence among hemodialysis patients and to explore potentially modifiable factors associated with low PB adherence.

**Methods:**

A cross-sectional study (502 patients) was performed in four dialysis units in Salvador, Brazil, using data from the second phase of the Prospective Study of the Prognosis of Chronic Hemodialysis Patients (PROHEMO). Patients were categorized as adherent or non-adherent to PB based on their responses to a semi-structured questionnaire.

**Results:**

Non-adherence to PB was observed for 65.7% of the patients. After adjustments for numerous covariates, cerebrovascular disease (odds ratio (OR), 3.30; 95% confidence interval (CI), 1.03-10.61), higher PTH (OR per each 300 pg/mL, 1.14; 95% CI, 1.01-1.28), lack of comprehension of the appropriate time to use PB (OR, 7.09; 95% CI, 2.10-23.95) and stopping PB use after feeling better (OR, 4.54; 95% CI, 1.45-14.25) or feeling worse (OR, 11.04; 95% CI, 1.79- 68.03) were significantly associated with PB non-adherence. By contrast, the adjusted odds of PB non-adherence were lower for patients with more years on dialysis (OR by each 2 years, 0.87; 95% CI, 0.80-0.95), with serum phosphorus above 5.5 mg/dL (OR, 0.53; 95% CI 0.34-0.82), who referred that were encouraged by the dialysis staff to be independent (OR, 0.52; 95% CI 0.30-0.90), and reported that the nephrologist explained how PB should be used (OR, 0.20; 95% CI 0.05-0.73).

**Conclusion:**

The results of the present study are encouraging by showing evidence that improvement in the care provided by the dialysis staff and the attending nephrologist may play an important role in reducing the high prevalence of non-adherence to PB in maintenance hemodialysis patients. A new questionnaire is presented and may help to evaluate systematically the patients regarding PB adherence in hemodialysis setting.

## Background

The deterioration of kidney function is accompanied by disturbances in mineral metabolism, including decreased vitamin D and calcium levels and increased parathyroid hormone (PTH) and phosphorus levels, which begin in the early stages of the disease [1]. In patients on dialysis, abnormalities in serum phosphorus, calcium and PTH levels have been shown to be associated with increased cardiovascular mortality [2-6]. Although all of these abnormalities must be controlled, phosphorus level control has received special attention because of the strong evidence linking hyperphosphatemia and cardiovascular mortality [2]. Therefore, to improve survival, current guidelines advocate more strict phosphorus control than in the past, and this control is usually achieved by optimizing renal replacement therapy, restricting dietary phosphorus intake and using a phosphorus binder [3,4].

For patients on renal replacement therapy, it is important to avoid non-adherence to the dialysis schedule and to adjust the parameters to improve phosphorus removal in hemodialysis therapy [7-10]. With regard to phosphorus intake, phosphorus restriction can result in protein restriction, and thus, attention is required to maintain an adequate protein intake [11]. A significant proportion of phosphorus intake often comes from the phosphate salts that are used as additives and preservatives, especially in processed and fast foods. Thus, dietary counseling to avoid food with high phosphorus content, while ensuring adequate protein intake, can help in the management of serum phosphorus concentration [8]. However, despite the use of optimized renal replacement therapy and adequate dietary phosphorus restriction, most patients must still use a phosphate binder to control their phosphorus levels [8]. It is important to note that there is evidence that phosphate binders are associated with longer survival because they allow for less severe protein restriction, and consequently, they are associated with improved nutritional status [12,13]. The use of a phosphate-restricted diet, in combination with an oral phosphate binder, has become well established in the management of patients on dialysis, although there is not yet consensus among experts regarding this. It is important to note that according to the K/DIGO Work Group, the strength of indication for prescribing phosphate binder to patients with CKD stage 5D is graded as Level 2 (suggested) and the evidence quality grade is B (moderate) [8,12,13].

Although the use of phosphate binders has become well established, many patients on dialysis do not take these drugs as prescribed. Therefore, the barriers to phosphate binder adherence should be considered. The use of phosphate binders is associated with side effects, most commonly of gastrointestinal origin, and these side effects can interfere with social habits and with individual lifestyles, and the use of phosphate binders also increases the burden of daily pills and treatment costs [8,14-16]. Studies have shown that the prevalence of non-adherence to phosphate binders varies from 22% to 74% (mean 51%) [16]. It is important to note that this large variability can be explained by the different methodologies used to measure adherence in these studies, with most of the studies inappropriately using the serum phosphorus level to measure adherence [16].

Given the necessity of phosphorus control and considering the benefits of the nutritional improvement possible with the use of phosphate binders by hemodialysis patients, as well as that the newer phosphate binders are very expensive, it is important to explore the barriers to phosphate binder adherence and to consider strategies to avoid non-adherence. The objectives of this study were to evaluate adherence to phosphate binders among hemodialysis patients and to explore the potentially modifiable factors that are associated with low phosphate binder adherence using a new structured questionnaire specially designed for the PROHEMO.

## Methods

The present cross-sectional study was performed using the baseline data of patients enrolled in the second phase of the Prospective Study of the Prognosis of Chronic Hemodialysis Patients (PROHEMO) from January 2010 to December 2010. The PROHEMO is an ongoing prospective cohort study of adult hemodialysis patients receiving treatment at four satellite dialysis units in the city of Salvador, BA, Brazil [17]. The Institutional Review Board of the Medical School of the Federal University of Bahia approved the study protocol, and all of the patients provided informed consent to participate.

Data on adherence to phosphate binders were obtained for 581 patients through face-to-face interviews, using a structured questionnaire specially designed for the PROHEMO (Additional file 1). A total of 502 patients reported that they had been prescribed a phosphate binder by a nephrologist, and this information was confirmed by reviewing their medical records. These 502 patients constituted the sample for this study.

The patients were categorized as adherents or non-adherents to phosphate binders. To be considered adherent, the patients had to report that they always used or almost always used the prescribed phosphate binder when consuming foods rich in phosphorus, such as milk, cheese, yogurt, meat, chicken, fish, beans, lentils, chickpeas, soybeans, peas, ham, sausage, chocolate and soda. Additionally, they had to report never or rarely missing taking the phosphate binder with a meal rich in phosphorus during the previous month.

The collection of demographic data, laboratory parameters, comorbidities and other clinical variables was performed as soon as the patients were enrolled in the study. The data were abstracted from medical records and were supplemented with information provided by the patient and the attending nephrologist. The laboratory values were based on the pre-dialysis measurements. The dialysis dose was determined by the single-pool Kt/V. To determine the economic class (A, B, C, D, E) of each patient, the classification system of the Brazilian Institute of Market Research (Abipeme) was used [18]. This classification takes into account consumer goods, such as refrigerators, televisions and telephones. Patients in classes D and E were categorized as poor or very poor [18]. The items used to calculate the generic score on the Portuguese version of the Kidney Disease Quality of Life Short Form (KDQOL-SF) were used to determine the mental component summary (MCS) and the physical component summary (PCS) of health-related quality of life [19]. The complete Portuguese version of the Center for Epidemiological Studies Depression Index (CES-D) was used to assess symptoms of depression [20].

The patients were asked about their understanding of the correct time to take the phosphate binders relative to the intake of foods rich in phosphorus, i.e., within 30 minutes after food intake or more than 30 minutes after food intake. Intentional lack of adherence due to the patients’ beliefs regarding the reasons for taking the medication was investigated by asking the patients if they had ever stopped taking the phosphate binder because they felt better or worse.

To assess social interaction and support, the patients’ responses to two separate questions were used. The patients were asked about the degree of satisfaction with the time that they were able to spend with family and friends and about the support that they received from family and friends. The responses to these questions could vary from “very dissatisfied” to “very satisfied.” Patients who responded “somewhat dissatisfied” or “very dissatisfied” were compared with those who responded “somewhat satisfied” or “very satisfied.” The patients were asked five questions to assess their interactions with the staff of the dialysis clinics. Two questions required a yes-or-no response based on the patient’s perception of the explanations provided by the dietitian and nephrologist regarding the correct use of the phosphate binder. The other three questions were aimed to evaluate the patient’s satisfaction with the care provided by the staff, the patient’s perception of the support provided by the staff or coping with the disease and the patient’s perception that he/she received encouragement from the staff to be independent as possible. The level of satisfaction with care was classified into three categories: “regular to very poor,” “good” and “very good to the best,” with “good” being the reference category. The patient’s perception of the support and encouragement provided by the staff was rated on a five-point Likert scale ranging, from “definitely true” to “definitely false.” Patients who responded “definitely true” or “mostly true” were compared with those who responded “don’t know,” “mostly false” or “definitely false.”

### Statistical analysis

The results are expressed as mean ± SD or as median and interquartile range (IQR) for continuous variables and as percentage for categorical variables. To test for differences between groups, the chi-square or Fisher’s exact test was used for categorical variables, and the *t*-test or the Mann–Whitney test was used for quantitative variables.

Two groups of adjusted logistic regression models were used to assess associations of non-adherence to the phosphate binder with patient’s characteristics, the understanding and beliefs of the patients about the use of the phosphate binder and personal interactions. The first group of adjusted logistic regression models (minimally adjusted models) included age, race, sex, education, economic class, living with family, marital status, years on dialysis and dialysis by catheter. The second group of models (extensively adjusted models) included cerebrovascular disease, diabetes, heart failure, ischemic heart disease, peripheral vascular disease, physician-diagnosed depression, depression symptom score, cancer, PTH, serum phosphorus, blood hemoglobin, albumin-corrected serum calcium, serum creatinine and the co-variables included in the minimally adjusted models. Binary indicator variables were used to address missing categorical covariate information. All of the analyses were performed using SPSS software, version 21 for Mac.

## Results

A total of 330 of the 502 patients (65.7%) were considered non-adherent to phosphate binders. The most common phosphate binders prescribed were sevelamer hydrochloride (277/502 patients, 55.2%) and calcium carbonate (159/502, 31.7%). A small number of patients were prescribed calcium acetate (5/502; 1%) or a combination of sevelamer hydrochloride and calcium carbonate (7/502; 1.4%). A total of 54 of 502 patients (10.8%) said that although a nephrologist had prescribed a phosphate binder, they did not use it, so they did not answer the question about which phosphate binder they were using.

Table 1 shows the characteristics of the patients by category of phosphate binder adherence. Compared with the group of adherents to phosphate binders, the group of non-adherents to phosphate binders had a marginally significantly higher percentage of men (66.3% vs. 57.6%, P =0.058), a younger mean age (46.98 ± 13.25 yr vs. 49.21 ± 13.26 yr, P =0.074) and a lower median number of months on dialysis (48.78 vs. 60.15, P =0.056). A significantly (P = 0.011) higher median PTH level was observed for non-adherent patients than for adherent patients (398.0 pg/mL [IQR = 150.70 pg/mL −726.60 pg/mL] vs. 252.20 pg/mL [IQR = 130.10 pg/mL-588.80 pg/mL]). The mean depression symptom scores determined by the CES-D were very similar between adherence categories (14.0 ± 10.7 for non-adherent patients and 13.6 ± 10.7 for adherent patients, P = 0.691). Small differences by adherence category were also observed for the means of Kt/V, PCS and MCS (data not shown in the table).

**Table 1:** Sociodemographic characteristics, comorbidities and laboratory values of the study patients (n = 502)

**Characteristics**	**Non-adherent (n = 330)**	**Adherent (n = 172)**	**P-value**
Age, years (mean ± SD)	46.98 ± 13.25	49.21 ± 13.26	0.074
% Non-white	90.6	87.8	0.326
% Male	66.3	57.6	0.058
Months on dialysis (median [IQR])	48.78 [24.66-96.26]	60.15 [30.44-118.83]	0.056
CESD-score (mean ± SD)	14.00 ± 10.70	13.58 ± 10.72	0.691
**% Social characteristics**			
Married	58.7	57	0.706
> High school	63.5	61.6	0.676
Working before dialysis	75.7	76.2	0.905
Working currently	17.3	12.8	0.186
Poor or very poor	48.9	41.5	0.116
Living with family	86.9	90.7	0.214
**% Comorbidities**			
Heart failure	8.6	12.2	0.197
Cerebrovascular disease	5.5	2.3	0.103
Diabetes	16.7	20.3	0.315
Physician-diagnosed depression	5.8	4.7	0.607
Peripheral vascular disease	3.3	4.1	0.669
Ischemic heart disease	9.4	9.9	0.868
Cancer	3.3	3.5	0.923
**% Vascular access catheter**	9.4	4.7	0.060
Hemoglobin, g/dL (mean ± SD)	10.49 ± 1.96	10.78 ± 1.62	0.096
Albumin, g/dL (mean ± SD)	3.74 ± 0.52	3.80 ± 0.36	0.232
^a^ Calcium, mg/dL (mean ± SD)	8.91 ± 0.84	8.94 ± 1.09	0.708
Phosphorus, mg/dL(mean ± SD)	5.46 ± 1.36	5.63 ± 1.52	0.185
PTH, pg/mL (median [IQR])	398.00 [150.70-726.60]	252.20 [130.10-588.80]	0.011
Ca x P product	48.73 ± 13.34	50.46 ± 15.30	0.190

^a^ calcium corrected for serum albumin.

*IQR* interquartile range.

Number with missing data: working before dialysis (1); working currently (1); very poor (6); living with family (1); heart failure (4); cerebrovascular disease (1); diabetes (1); depression (2); peripheral vascular disease (2); ischemic heart disease (1); cancer (2); PTH (4).

Almost all of the adherent patients to phosphate binders (169/172) and approximately 80% (257/330) of those who were non-adherents to phosphate binders reported that a doctor had explained how to use the medication (P >0.001). Almost 70% (119/172) of the adherent patients and almost 60% (84/330) of the non-adherent patients said that a dietitian had explained how to use the medication (P >0.001).

More than a half of the patients studied (264/502) never took the phosphate binder when eating hemodialysis snacks, although most of them (85%; 426/502) ate foods rich in phosphorus as hemodialysis snacks. Almost 30% (145/502) of the patients said that they usually forgot to take the phosphate binder with snacks.

Regarding the comprehension of phosphate binder use, almost 90% (437/502) of the patients said that they did not take the phosphate binder when eating foods with very low phosphorus content. Approximately 40% of the patients (195/502) thought that they were supposed to take the phosphate binder when eating foods rich in phosphorus, 43.62% (219/502) thought that they had to take it with their main meals (independent of the phosphorus intake), and 6.77% (34/510) of the patients said that they did not understand when to take the phosphate binder. In univariate analysis, none of the patients in the adherent group (0/172) and 10% (34/330) in the non-adherent group (P >0.001) did not understand how to use the phosphate binder.

To assess another comprehension item of phosphate binder use the patient was asked about the time to take the medication after a meal rich in phosphorus. In the univariate analysis, more than 60% of the patients in the adherent group (107/172) said that they never took the phosphate binder more than 30 minutes after such a meal, and almost half of the patients (155/330) in the non-adherent group said the same thing (P >0.001).

Almost 15% (31/216) of patients who were non-adherents and approximately 6% (9/153) of the adherents to the phosphate binder therapy reported that they always or almost always stopped taking the phosphate binder on their own initiative after feeling worse (P =0.031). In addition, 9.5% (25/264) of non-adherent patients and almost 2.5% (4/171) of adherents reported that they always or almost always stopped taking the phosphate binder on their own initiative after feeling better (P >0.001). The patients were also asked whether they had stopped taking the phosphate binder because they did not have the medication; almost 12% (39/330) of non-adherent patients and almost 10% (16/172) of adherents reported that they always or almost always stopped taking the phosphate binder for this reason (P >0.001). No difference between the adherents and non-adherents was observed in the univariate analysis with regard to stopping phosphate binder use on one’s own initiative associated with any specific symptom (P =0.510).

Table 2 shows the adjusted odds ratios for the associations between patient characteristics and non-adherence to the phosphate binder. More years on dialysis and serum phosphorus >5.5 mg/dL (compared to 3.5-5.5 mg/dL) were associated with lower odds of non-adherence to the phosphate binder in both the minimally and extensively adjusted logistic regression models. In the extensively adjusted model, greater odds of non-adherence were observed for patients with higher PTH levels and cerebrovascular disease.

**Table 2:** Adjusted odds ratios for the associations between patient characteristics and non-adherence to phosphate binder use

**Patient characteristics**	**Odds ratios (95%Confidence intervals)**	
	**Minimally adjusted**	**Extensively adjusted**
**Sociodemographics**		
Age per each 10 years	0.88 (0.76-1.02)	0.87 (0.73-1.04)
Female vs. male	1.53 (1.03-2.29)	1.48 (0.94-2.33)
Non-white vs. white	1.24 (0.66-2.33)	1.28 (0.67-2.47)
Less than high school vs higher	1.09 (0.71-1.70)	1.15 (0.73-1.82)
Poor/very poor vs. higher economic class	1.20 (0.78-1.84)	1.23 (0.78-1.94)
Married (yes vs. no)	1.09 (0.72-1.64)	1.07 (0.70-1.65)
Living with family (yes vs. no)	0.65 (0.34-1.26)	0.64 (0.32-1.27)
Years on dialysis (each 2 years)	0.91(0.84-0.99)	0.87 (0.80-0.95)
Catheter as vascular access (yes vs. no)	1.87 (0.82-4.28)	2.10 (0.85-5.15)
**Comorbid conditions (yes vs. no)**		
Cerebrovascular disease	2.86 (0.93-8.77)	3.30 (1.03-10.61)
Diabetes	0.69 (0.41-1.18)	0.71 (0.40-1.29)
Heart failure	0.77 (0.41-1.44)	0.84 (0.43-1.65)
Ischemic heart disease	0.99 (0.52-1.93)	1.06 (0.52-2.20)
Peripheral vascular disease	0.82 (0.30-2.26)	0.87 (0.29-2.63)
Physician-diagnosed depression	1.15 (0.47-2.80)	1.17 (0.46-3.01)
Depression score by CES-D* ≥18 vs. lower	0.88 (0.57-1.37)	0.90 (0.57-1.44)
Cancer	1.03 (0.36-2.96)	1.09 (0.37-3.23)
**Laboratory variables**		
PTH (per 300 pg/mL higher)	1.11 (0.99-1.24)	1.14(1.01-1.28)
Serum phosphorus (>3.5 vs. 3.5-5.5 mg/dL)	0.43 (0.19-0.93)	0.50 (0.22-1.13)
Serum phosphorus (>5.5 vs. 3.5-5.5 mg/dL)	0.50 (0.33-0.75)	0.53 (0.34-0.82)
Serum calcium (per 1 mg/dL higher)	0.99 (0.81-1.22)	1.02 (0.82-1.27)
Hemoglobin (>11 vs. ≥11 g/dL)	0.98 (0.67-1.45)	0.94 (0.62-1.41)
Serum albumin (>3.5 vs. ≥3.5 g/dL)	1.48 (0.93-2.35)	1.50 (0.91-2.50)
Serum creatinine (per 1 mg/dL higher)	0.95 (0.89-1.00)	0.97 (0.91-1.04)

*CES-D* Center for Epidemiological Studies Depression Scale.

Minimally adjusted odds ratios were adjusted for age, sex, race, education, economic class, marital status, living with family, years on dialysis and dialysis by catheter.

Extensively adjusted odds ratios were adjusted for all listed variables.

Table 3 shows the minimally and extensively adjusted logistic regressions for the association of the odds of non-adherence to the phosphate binder with the factors deemed potentially modifiable, i.e., the patient’s understanding of PB use, the patient’s beliefs about PBs, not having the medication, interactions between patients and their families and interactions between patients and the staff.

**Table 3:** Adjusted associations of patient’s attitudes and beliefs with non-adherence to phosphate binder use

**Patient’s attitudes and beliefs**	**Odds ratio**^**a**^**of Non-adherence(95%Confidence interval)**	
	**Minimally adjusted**^**a**^	**Extensively adjusted**^**a**^
**Patient’s attitude regarding or understanding of the time to take the phosphate binder**		
Rarely or never take the PB > 30 minutes after meals	Ref = 1	Ref = 1
Frequently take the PB > 30 minutes after meals	0.94 (0.52-1.68)	0.87 (0.47-1.61)
Always or almost always take the PB >30 minutes after meals	5.98 (2.03-17.58)	6.15 (2.04-18.50)
**Patient’s beliefs about stopping PB use after feeling better**		
Rarely or never stopped	Ref = 1	Ref = 1
Frequently stopped	1.57 (0.61-4.02)	1.47 (0.55-3.91)
Always or almost always stopped	4.21 (1.41-12.54)	4.54 (1.45-14.25)
**Patient’s beliefs about stopping PB use after feeling worse**		
Rarely or never stopped	Ref = 1	Ref = 1
Frequently stopped	0.82 (0.30-2.27)	0.66 (0.23-1.91)
Always or almost always stopped	7.94 (1.80-35.06)	7.26 (1.62-32.55)
**Patient’s report of stopping PB used because he/she did not have the medication**		
Rarely or never stopped	Ref = 1	Ref = 1
Frequently stopped	1.35 (0.68-2.68)	1.29 (0.63-2.65)
Always or almost always stopped	3.57 (0.75-17.01)	2.77 (0.57-13.47)
**Patient-family interactions**		
^b^Satisfied with time spent with family and friends	0.97 (0.60-1.57)	1.05 (0.62-1.77)
^b^Satisfied with support provided by family and friends	0.89 (0.49-1.61)	0.83 (0.44-1.58)
**Patient-staff interactions**		
^c^ Satisfaction with the care provided by the hemodialysis staff		
Good	Ref = 1	Ref = 1
Very poor to regular	1.11 (0.60-2.04)	1.25 (0.65-2.38)
Very good to the best	1.06 (0.69-1.63)	1.13 (0.71-1.80)
^d^Dialysis staff improved the patient’s ability to cope with the disease	0.62 (0.25-1.50)	0.67 (0.26-1.70)
^d^Dialysis staff encouraged the patient to be independent	0.52 (0.32-0.87)	0.52 (0.30-0.90)
^e^Nephrologist explained PB use	0.22 (0.62-0.78)	0.20 (0.05-0.73)
^e^Dietitian explained PB use	0.96 (0.62-1.47)	1.11 (0.69-1.79)

*PB* phosphate binder.

^a^Minimally adjusted odds ratios were adjusted for age, race, sex, education, economic class, living with family, marital status, years on dialysis and dialysis by catheter. Extensively adjusted odds ratios were adjusted for cerebrovascular disease, diabetes, heart failure, ischemic heart disease, peripheral vascular disease, physician-diagnosed depression, depression score, cancer, PTH, albumin-corrected serum calcium, serum phosphorus, blood hemoglobin, serum creatinine and all of the co-variables included in the minimally adjusted model.

^b^Patient responses were unsatisfied and very unsatisfied compared with satisfied and very satisfied.

^c^ Patient response was very poor to regular compared with very good to the best.

^d^ Patient response was definitely true or mostly true (positive responses) compared with do not know and mostly or definitely false.

^e^ Patient response was yes (positive response) compared with no.

### Patients’ attitudes about and understanding of the appropriate time to take the phosphate binder

The use of a phosphate binder more than 30 minutes after eating foods rich in phosphorus (always or almost always compared with rarely or never) was significantly associated with phosphate binder non-adherence in both the minimally adjusted model (OR, 5.98; 95% confidence interval (CI), 2.03-17.58) and the extensively adjusted model(OR, 6.15; 95% CI, 2.04-18.50). There was no difference in the frequency of non-adherence between patients who did not understand or thought that the phosphate binder should be used after the main meals and the patients who thought that the drug should be used after phosphorus intake in the minimally (OR, 1.18; 95%CI, 0.78-1.77) or extensively adjusted logistic regression models (OR, 1.19; 95% CI, 0.76-1.85) (data not shown in Table 3). The analysis comparing the patients who did not understand phosphate binder use and those who believed that they should take the drug after main meals was inaccurate because of the small percentage of patients who did not understand in both groups. It should be noted that in the adherent group, no patients reported not understanding.

### Patient’s beliefs

There was an association between phosphate binder non-adherence and stopping the phosphate binder after feeling better (always or almost always compared with rarely or never) in the minimally (OR, 4.21; 95% CI, 1.41-12.54) and extensively adjusted logistic regression models (OR, 4.54; 95% CI, 1.45-14.25). Stopping phosphate binder use after feeling worse (always or almost always compared with rarely or never) was significantly associated with phosphate binder non-adherence in both the minimally (OR, 7.94; 95% CI, 1.80-35.06) and extensively adjusted models (OR, 7.26; 95% CI, 1.62-32.55).

### Patient reports of stopping the use of phosphate binder because they did not have the medication

As shown in Table 3, patients non-adherent to phosphate binder (PB) were more likely to report that they stopped the use of PB because they did not have the medication, but the difference was not statistically significant. The extensively adjusted odds of a patients refer that stopped phosphate binder always or almost always (compared with rarely or never) because he/she did not have the medication were approximately 2.8 times higher in the group of non-adherents than in the group of adherents to phosphate binder (OR, 2.77; 95% CI, 0.57-13.47).

### Patient-family interactions

There was no statistically significant association between the patient’s satisfaction with the time spent with family and friends (very satisfied or satisfied versus unsatisfied or very unsatisfied) and non-adherence to phosphate binder use in either the minimally (OR, 0.97; 95% CI, 0.60-1.57) or extensively adjusted models (OR, 1.05; 95% CI, 0.62-1.77). Weak and statistically non-significant association with non-adherence to phosphate binder was observed for satisfaction with the support of family and friends (very satisfied or satisfied versus unsatisfied or very unsatisfied) in the minimally (OR, 0.89; 95% CI, 0.49-1.61) and extensively adjusted logistic regression models (OR, 0.83; 95% CI, 0.44-1.58).

### Patient-staff interaction

There was also no statistically significant association between satisfaction with the care provided by the hemodialysis staff (good, very good, excellent and the best versus very bad, bad and fair) and non-adherence to phosphate binder use in the minimally (OR, 1.06; 95% CI, 0.69-1.63) or extensively adjusted logistic regression models (OR, 1.13; 95% CI, 0.71-1.80). The belief that the dialysis staff supported the patients with coping with the disease (definitely true or mostly true versus do not know, mostly false or definitely false) was weakly associated (not statistically significant) with lower odds of non-adherence in the minimally (OR, 0.62; 95% CI, 0.25-1.50) and extensively adjusted logistic regression models (OR, 0.67; 95% CI, 0.26-1.70). The belief that the dialysis staff encouraged the patient’s independence (definitely true or mostly true versus do not know, mostly false or definitely false) was significantly associated with lower odds of non-adherence in both the minimally (OR, 0.52; 95% CI, 0.32-0.87) and extensively adjusted models (OR, 0.52; 95% CI, 0.30- 0.90). Having a nephrologist explain the use of the phosphate binder (yes versus no) was strongly associated with lower odds of non-adherence in both the minimally (OR, 0.22; 95% CI, 0.62-0.78) and extensively adjusted models (OR, 0.20; 95% CI, 0.05-0.73). There was no association between having a dietitian explain PB use (yes versus no) and phosphate binder non-adherence in the minimally (OR, 0.96; 95% CI, 0.62-1.47) or extensively adjusted logistic regression models (OR, 1.11; 95% CI, 0.69-1.79).

## Discussion

Similar to previous studies, this study showed that only a small number, approximately one-third, of the patients studied were adherent to the phosphate binder regimen [16]. It is possible that this low adherence rate was associated with the phosphate binder having to be taken during meals rich in phosphorus, interfering with social habits and individual lifestyles and increasing the burden of daily pills [14,15].

Karamanidou et al. found a high prevalence of phosphate binder non-adherence. In their systematic review, the percentage of non-adherent patients ranged from 22% to 74% (mean 51%) [16]. This large variability can be explained by the different methodologies used to measure adherence (such as self-reports, the serum phosphorus level, pill counts and others) and by the various definitions of adherence used in these studies [16]. Furthermore, this systematic review also showed that when adherence was assessed using the serum phosphorus level, the median percentage of patients considered non-adherent was 58%, but that this was 31% when assessed by self-reports [16].

However, the percentage of patients considered non-adherent in our study, assessed by self-reports, was higher than the median reported above. It is important to note that in our study, adherence was defined based on two different questions asked at different times during the interview, to avoid incorrect interpretations and to test and confirm that the patient had really understood the questions. This design might have been responsible for the higher non-adherence rate.

Another aim of our study was to expand the understanding of the potentially modifiable factors that are associated with non-adherence to phosphate binder use. Our study calls attention to several factors that are related to the patients themselves and to patient-dialysis staff interactions, rather than social, demographic, laboratory or clinical factors. Our study revealed that health beliefs, knowledge/understanding of medication use and the patient’s perception that the nephrologist had explained phosphate binder use and that the hemodialysis staff had encouraged them to be independent were associated with phosphate binder adherence.

Health beliefs are related to patients’ perceptions (“feelings”) about a medication, such as the potential adverse effects and benefits of taking it [14-16,21]. Although no specific symptoms from the PROHEMO adherence questionnaire were associated in our study with non-adherence, stopping phosphate binder use on one’s own initiative after “feeling better” or “feeling worse” was associated with non-adherence. It is possible that a greater focus on patient-dialysis staff interactions could eliminate the barriers, doubts and myths related to phosphate binder use and that the treatment benefits could be enhanced by improving adherence in this manner. Other studied factors could reinforce the importance of patient-dialysis staff interactions in determining phosphate binder adherence. These factors include knowledge/understanding of medication use and the patient’s perception that the nephrologist has explained phosphate binder use and that the hemodialysis staff has encouraged the patient to be independent.

Most of the sociodemographic factors assessed in our study were not associated with adherence to phosphate binders. Some studies have shown that older age is associated with higher levels of adherence [16,21]. In our study, age was not associated with adherence in multivariate analysis, although univariate analysis showed a marginally significant association between older age and adherence.

None of the laboratory variables, except for the phosphorus and PTHi levels, were associated with adherence to phosphate binders. The extensively adjusted multivariate analysis showed that phosphorus levels greater than 5.5 mg/dL, compared with levels in the range of 3.5-5.5 mg/dL, were associated with adherence to phosphate binders. Thus, patients with higher phosphorus levels seem to be more adherent if the phosphorus level and adherence are studied at the same time point. This association might only reflect that patients who have higher phosphorus levels use phosphate binders because they are strongly advised to do so or that patients who use phosphate binders feel freer to eat more foods that are rich in phosphorus, thus requiring better dietary counseling. We could not determine the cause of this association because our study did not evaluate daily phosphorus intake. Higher PTHi levels were also associated with non-adherence, and this association most likely reflects secondary hyperparathyroidism as a consequence of permanent higher levels of phosphorus in these patients [22].

Two other hypotheses assessed in our study were that poor health-related quality of life and symptoms of depression would be associated with phosphate binder non-adherence, but we did not find evidence supporting these hypotheses. Regarding clinical variables, comorbidities, except for cerebrovascular disease, were also not associated in our study with non-adherence. It is possible that cerebrovascular disease was associated with non-adherence because cerebrovascular disease can reduce a patient’s autonomy and the independence necessary to self-medicate, which appears to be important in phosphate binder adherence. More time on hemodialysis was associated with better adherence, possibly reflecting the importance of time in patient-dialysis staff interactions in terms of patients’ acceptance and knowledge of the disease and therapy.

Moreover, the investigated social aspects were not associated in our study with phosphate binder adherence. In Brazil, the government usually provides sevelamer hydrochloride, and in some cities, such as Salvador, calcium carbonate is also provided, and it appears that not having the medicine is more directly related to the level of government, personal or family organization than to the patients’ ability to afford the drug.

Our study had some limitations. One limitation was the methodology and its subjectivity, due to self-reports and interviewer interpretations. To the best of our knowledge, there are no validated questionnaires available to evaluate the degree of adherence to phosphate binders and one of the contributions of this study is a new applicable questionnaire that can be used in future research. Because the interviews were conducted by only one researcher, different interpretations were at least minimized. Another factor was the cross-sectional nature of the study, which made it impossible to establish cause-and-effect relationships for the observed associations. Other relevant factors were that the patients’ preferred phosphate binders were not determined for the completely non-adherent patients, and the tablets’ tastes and sizes were not objectively described in the questionnaire, although they could be barriers to phosphate binder adherence. Our study did not explore the personality characteristics and regimen complexity that might be important in determining adherence to phosphate binder therapy [23].

Additional high-quality studies are needed to explore barriers to phosphate binder adherence in greater detail and we expect that the questionnaire used, which was developed to provide a comprehensive assessment of adherence to phosphate binder by hemodialysis patient, will stimulate new investigations and more systematic assessment of phosphate binder adherence in hemodialysis setting.

To the best of our knowledge, the sample size used in this study was the largest used to date to assess phosphate binder adherence based on self-reports. Although a large sample size is important, it is possible that the results were influenced by Brazil’s health care system providing both of the most commonly prescribed phosphate binders (calcium carbonate and sevelamer hydrochloride). Therefore, it is not possible generalize these results to other health care systems.

## Conclusion

Our study suggests that almost 70% of hemodialysis patients are phosphate binder non-adherent, and if the health care system provides the medication, economic aspects seem not to be associated with non-adherence. It appears that some of the identified potentially modifiable factors can be improved by optimizing the attention paid to patients by the staff. A new questionnaire was proposed and the knowledge gained by the responses provided by the patients should be useful to guide interventions aimed at improving adherence and ensure the correct use of the medication.

## Abbreviations

CES-D: Center for epidemiological studies depression index; CI: Confidence interval; KDQOL-SF: Kidney disease quality of life short form; MCS: Mental component summary; OR: Odds ratio; PB: Phosphate binder; PCS: Physical component summary; PROHEMO: Prospective study of the prognosis of chronic hemodialysis patients; PTH: Parathyroid hormone.

## Competing interests

The authors declare that they have no competing interests.

## Authors’ contributions

MTSM: Literature review, data collection and first draft of the paper. LFS: Data collection and comments on the drafts. AK: Data collection and comments on the drafts. DR: Data collection and comments on the drafts, LD: Data collection and comments on the drafts. GS: Data collection and comments on the drafts. LO: Data collection and comments on the drafts. GBL: Data collection and comments on the drafts. AAL: Principal investigator in the PROHEMO study. Designed the study, structured the paper, performed the statistical analysis and reviewed several versions of the manuscript. All authors read and approved the final manuscript.

## Pre-publication history

The pre-publication history for this paper can be accessed here:

http://www.biomedcentral.com/1471-2369/14/208/prepub

## Supplementary Material

Additional file 1 Phosphate Binder Adherence PROHEMO Questionnaire. Click here for file

## References

[B1] Zehnder D Landray MJ Wheeler DC Fraser W Blackwell L Nuttall S Hughes SV Townend J Ferro C Baigent C Hewison M Cross-sectional analysis of abnormalities of mineral homeostasis, vitamin D and parathyroid hormone in a cohort of pre-dialysis patients. The chronic renal impairment in Birmingham (CRIB) study Nephron Clin Pract 2007 107 c109 c116 10.1159/000108652 17890873

[B2] Covic A Kothawala P Bernal M Robbins S Chalian A Goldsmith D Systematic review of the evidence underlying the association between mineral metabolism disturbances and risk of all-cause mortality, cardiovascular mortality and cardiovascular events in chronic kidney disease Nephrol Dial Transplant 2009 24 1506 1523 10.1093/ndt/gfn613 19001560

[B3] Block AG Hulbert-Shearon TE Levin NW Port FK Association of serum phosphorus and calcium × phosphate product with mortality risk in chronic haemodialysis patients: a national study Am J Kidney Dis 1998 31 607 617 10.1053/ajkd.1998.v31.pm9531176 9531176

[B4] Young EW Albert JM Satayathum S Goodkin DA Pisoni RL Akiba T Akizawa T Kurokawa K Bommer J Piera L Port FK Predictors and consequences of altered mineral metabolism: the Dialysis Outcomes and Practice Patterns Study Kidney Int 2005 67 1179 1187 10.1111/j.1523-1755.2005.00185.x 15698460

[B5] Block GA Klassen PS Lazarus JM Ofsthun N Lowrie EG Chertow GM Mineral metabolism, mortality and morbidity in maintenance hemodialysis J Am Soc Nephrol 2004 15 2208 2218 10.1097/01.ASN.0000133041.27682.A2 15284307

[B6] Ganesh SK Stack AG Levin NW Hulbert-Shearon T Port FK Association of elevated serum PO4, CA X PO4 product and parathyroid hormone with cardiac mortality risk in chronic hemodialysis patients J Am Soc Nephrol 2001 12 2131 2138 11562412 10.1681/ASN.V12102131

[B7] Molony DA Stephens BW Derangements in phosphate metabolism in chronic kidney diseases/endstage renal disease: therapeutic considerations Adv Chronic Kidney Dis 2011 18 120 131 10.1053/j.ackd.2011.02.004 21406297

[B8] Kidney Disease Improving Global Outcomes (KDIGO) CKD-MBD Work Group KDIGO clinical practice guideline for the diagnosis, evaluation, prevention, and treatment of chronic kidney disease-mineral and bone disorder (CKD-MBD) Kidney Int Suppl 2009 113 Suppl 1 130 10.1038/ki.2009.188 19644521

[B9] Gotch FA Panlilio F Sergeyeva O Rosales L Folden T Kaysen G Levin NW A kinetic model of inorganic phosphorus mass balance in hemodialysis therapy Blood Purif 2003 21 51 57 10.1159/000067866 12566662

[B10] Tonelli M Wang W Hemmelgarn B Lloyd A Manns B Alberta Kidney Disease Network Phosphate removal with several thrice weekly dialysis methods in overweight hemodialysis patients Am J Kidney Dis 2009 54 1108 1115 10.1053/j.ajkd.2009.05.018 19619920

[B11] Shinaberger CS Kilpatrick RD Regidor DL McAllister CJ Greenland S Kopple JD Kalantar-Zadeh K Longitudinal associations between dietary protein intake and survival in hemodialysis patients Am J Kidney Dis 2006 16 29 33 10.1053/j.ajkd.2006.03.049 16797385

[B12] Isakova T Gutiérrez OM Chang Y Shah A Tamez H Smith K Thadhani R Wolf M Phosphorus binders and survival on hemodialysis J Am Soc Nephrol 2009 20 388 396 10.1681/ASN.2008060609 19092121 PMC2637053

[B13] Lopes AA Tong L Thumma J Li Y Fuller DS Morgenstern H Bommer J Kerr PG Tentori F Akiba T Gillespie BW Robinson BM Port FK Pisoni RL Phosphate binder use and mortality among hemodialysis patients in the Dialysis Outcomes and Practice Patterns Study (DOPPS): evaluation of possible confounding by nutritional status Am J Kidney Dis 2012 60 90 101 10.1053/j.ajkd.2011.12.025 22385781 PMC3901075

[B14] Arenas MD Malek T Álvarez-Ude F Gil MT Moledous A Reig-Ferrer A Phosphorus binders: preferences of patients on haemodialysis and its impact on treatment compliance and phosphorus control Nefrologia 2010 30 522 530 20613851 10.3265/Nefrologia.pre2010.may.10275

[B15] Chiu YW Teitelbaum I Misra M de Leon EM Adzize T Mehrotra R Pill burden, adherence, hyperphosphatemia, and quality of life in maintenance dialysis patients Clin J Am Soc Nephrol 2009 4 1089 1096 10.2215/CJN.00290109 19423571 PMC2689877

[B16] Karamanidou C Clatworthy J Weinman J Horne R A systematic review of the prevalence and determinants of nonadherence to phosphate binder medication in patients with end-stage renal disease BMC Nephrol 2008 9 2 10.1186/1471-2369-9-2 18237373 PMC2270809

[B17] Martins MT Silva LF Martins MT Matos CM Melo NA Azevedo MF Travessa IE Amoedo MK Fernandes PA Nogueira FC Lopes GB Lopes AA Prescription of phosphorus binders and calcitriol for chronic hemodialysis patients Rev Assoc Med Bras 2009 55 70 74 19360282 10.1590/s0104-42302009000100019

[B18] Associação Brasileira de Institutos de Pesquisa de Mercado Pesquisa do perfil sócio-econômico e cultural do estudante de graduação das IFES brasileiras: classificação socioeconômica critério Abipeme http://www.ufrn.br/sites/fonaprace/perfil_anexo3.doc

[B19] Duarte PS Miyazaki MCQS Ciconelli RM Sesso R Translation and cultural adaptation of the quality of life assessment instrument for chronic renal patients (KDQOL-SF)] Rev Assoc Med Bras 2003 49 375 381 10.1590/S0104-42302003000400027 14963588

[B20] Silveira DX Jorge MR Propriedades psicométricas da escala de rastreamento populacional para depressão CES-D em populações clínica e não clínica de adolescentes e adultos jovens Psiq Clin 1998 25 251 261

[B21] Stamatakis MK Pecora PG Gunel E Factors influencing adherence in chronic dialysis patients with hiperphosphatemia J Renal Nutr 1997 7 144 148 10.1016/S1051-2276(97)90065-0

[B22] National Kidney Foundation K/DOQI clinical practice guidelines for bone and mineral metabolism and disease in chronic kidney disease Am J Kidney Dis 2003 42 Suppl 3 1 201 14520607

[B23] Wang S Alfieri T Ramakrishnan K Braunhofer P Newsome BA Serum phosphorus levels and pill burden are inversely associated with adherence in patients on hemodialysis Nephrol Dial Transplant 2013 0 1 9 10.1093/ndt/gft280 PMC4209875 24009281

