# Supplementary material for: Potentially modifiable factors associated with non-adherence to phosphate binder use in patients on hemodialysis
Source: BMC Nephrol. 2013 Oct 3;14:208. doi: 10.1186/1471-2369-14-208 (PMC3851130; doi:10.1186/1471-2369-14-208)
Supplement: Additional file 1 — Phosphate Binder Adherence PROHEMO Questionnaire. [file 1471-2369-14-208-S1.docx]

**Additional file 1:** Phosphate Binder Adherence PROHEMO Questionnaire

| 1. **Has your doctor prescribed medication to reduce the phosphorus level (chelating)?** 2. **Was a phosphate binder prescribed according to the medical records? (interviewer must check the records)** 3. **Did your doctor provide instructions on how to take the medicine to lower the phosphorus level (chelating)?** 4. **Did a dietitian provide instructions on how to take the medicine to lower the phosphorus level (chelating)?** 5. **What is your understanding of how the phosphate binder should be used?**    1. No understanding    2. With main meals (breakfast, lunch, dinner)    3. According to phosphorus intake, at the same time as foods rich in phosphorus    4. Does not apply 6. **How many times do you eat per day?** 7. **Which phosphate binder was prescribed?** 8. **How often do you use a phosphate binder when you consume these phosphorus-rich foods (milk, cheese, yogurt, meat, chicken, fish, beans, lentils, chickpeas, soybeans, peas, ham, sausage, chocolate, soda)?**    1. Always    2. Almost always    3. Frequently    4. Rarely    5. Never    6. Does not apply 9. **At which meal(s) do you always take the phosphate binder? (According to the prescription)** 10. **At which meal(s) do you always forget to take the phosphate binder? (According to the prescription).** 11. **If you eat something rich in phosphorus during hemodialysis, do you take the phosphate binder?**     1. Always     2. Almost always     3. Frequently     4. Rarely     5. Never     6. Does not apply 12. **Do you take the phosphate binder when you consume fruits, juices, black coffee or waffles?**   12.1 Always  12.2 Almost always  12.3 Frequently  12.4 Rarely  12.5 Never  12.6 Does not apply | 1. **Have you forgotten to take the phosphate binder after a meal rich in phosphorus (milk, cheese, yogurt, meat, chicken, fish, beans, lentils, chickpeas, soybeans, peas, ham, sausage, chocolate, soda) sometime during the last month?**    1. Always    2. Almost always    3. Frequently    4. Rarely    5. Never    6. Does not apply 2. **Have you used the phosphate binder more than 30 minutes after the ingestion of foods rich in phosphorus (milk, cheese, yogurt, meat, chicken, fish, beans, lentils, chickpeas, soybeans, peas, ham, sausage, chocolate, soda) sometime during the last month?**    1. Always    2. Almost always    3. Frequently    4. Rarely    5. Never    6. Does not apply 3. **Have you ever stopped taking the phosphate binder because you felt better?**     1. Always    2. Almost always    3. Frequently    4. Rarely    5. Never    6. Does not apply 4. **Have you ever stopped taking the phosphate binder on your own initiative after feeling worse?**    1. Always    2. Almost always    3. Frequently    4. Rarely    5. Never    6. Does not apply 5. **Which symptoms do you associate with the use of the phosphate binder?**    1. Bloating    2. Nausea or vomiting    3. Diarrhea    4. Constipation    5. Change in taste    6. Others: (open question)    7. Does not apply 6. **Have you ever stopped taking the phosphate binder because you did not have the medication?**    1. Always    2. Almost always    3. Frequently    4. Rarely    5. Never    6. Does not apply |
| --- | --- |
